# Prospective validation of the EuroSCORE II risk model in a single Dutch cardiac surgery centre

**DOI:** 10.1007/s12471-018-1161-x

**Published:** 2018-09-19

**Authors:** E. K. Hogervorst, P. M. J. Rosseel, L. M. G. van de Watering, A. Brand, M. Bentala, B. J. M van der Meer, J. G. van der Bom

**Affiliations:** 1 Centre for Clinical Transfusion Research, Sanquin Research, Leiden, The Netherlands; 2 Jon J van Rood Centre for Clinical Transfusion Research, Leiden University Medical Centre, Leiden, The Netherlands; 3 Department of Anaesthesia and Intensive Care, Amphia Hospital, Breda, The Netherlands; 4 TIAS, Tilburg University, Tilburg, The Netherlands; 5 Department of Clinical Epidemiology, Leiden University Medical Centre, Leiden, The Netherlands; 6 Department of Anaesthesiology, University of Groningen, Groningen, The Netherlands; 7 University Medical Centre Groningen, Groningen, The Netherlands

**Keywords:** Cardiac surgery, EuroSCORE, Risk model

## Abstract

**Objective:**

The EuroSCORE I was one of the most frequently used pre-operative risk models in cardiac surgery. In 2011 it was replaced by its successor the EuroSCORE II. This study aims to validate the EuroSCORE II and to compare its performance with the EuroSCORE I in a Dutch hospital.

**Methods:**

The EuroSCORE II was prospectively validated in 2,296 consecutive cardiac surgery patients between 1 April 2012 and 1 January 2014. Receiver operating characteristic curves on in-hospital mortality were plotted for EuroSCORE I and EuroSCORE II, and the area under the curve was calculated to assess discriminative power. Calibration was assessed by comparing observed versus expected mortality. Additionally, analyses were performed in which we stratified for type of surgery and for elective versus emergency surgery.

**Results:**

The observed mortality was 2.4% (55 patients). The discriminative power of the EuroSCORE II surpassed that of the EuroSCORE I (area under the curve EuroSCORE II 0.871, 95% confidence interval (CI) 0.832–0.911; area under the curve additive EuroSCORE I 0.840, CI 0.798–0.882; area under the curve logistic EuroSCORE I 0.761, CI 0.695–0.828). Both the additive and the logistic EuroSCORE I overestimated mortality (predictive mortality additive EuroSCORE I median 5.0%, inter-quartile range 3.0–8.0%; logistic EuroSCORE I 10.7%, inter-quartile range 5.8–13.9), while the EuroSCORE II underestimated mortality (median 1.6%, inter-quartile range 1.0–3.5). In most stratified analyses the EuroSCORE II performed better.

**Conclusion:**

Our results show that the EuroSCORE II produces a valid risk prediction and outperforms the EuroSCORE I in elective cardiac surgery patients.

## What’s new?

- The EuroSCORE I, developed in 1999, has been an important risk model in cardiac surgery; its successor, EuroSCORE II, was presented in 2012 but has not been validated in a Dutch cohort
- We performed a validation study to assess whether or not the EuroSCORE II was a valid risk model in a Dutch hospital
- The EuroSCORE II outperformed its predecessor in a Dutch single-centre validation study, with the exception of emergency surgery

## Introduction

In a growing population of patients undergoing cardiac surgery (including older and more vulnerable patients), an accurate pre-operative risk assessment has become indispensable [1]. An often-used method for risk assessment in cardiac surgery is the European System for Cardiac Operative Risk Evaluation, better known as the EuroSCORE (ES). The first ES became available in 1999 and provided a simple additive and logistic risk calculation model based on European adult cardiac surgery patients, and was widely implemented [2]. The EuroSCORE I (ESI) was validated in the Netherlands for both short- and long-term mortality and morbidity [3]. Over time, it became clear that the ESI overestimated the 30-day and 90-day mortality risk [4, 5]. This overestimation was caused by improvements in peri-operative patient care, substantially reduced mortality rates and evoked the need for a renewed risk model [1].

In 2011 the successor of ESI, the EuroSCORE II (ESII), was presented. As with all new risk models it is important to externally validate this model in patients other than the sampled patient population from which the risk model was developed [6]. Differences in patient populations influence the performance of risk models and determine whether or not the model is fit for use in a particular population [7].

The validation studies that have been published so far show ambiguous results when comparing the ESII with other risk models like the ESI and the Society of Thoracic Surgery (STS) score [8, 9]. Furthermore, several studies used data which were collected before the ESII was developed, and several studies validated the study for surgical procedures for which the ESII was not intended [10–12].

In 2003 we performed the first study validating the ESI in cardiac surgery patients in the Netherlands [3]. As a sequel, the present study aims to validate the ESII risk model in patients undergoing cardiac surgery in the Netherlands and also to compare ESII performance with the performance of the additive and logistic ESI.

## Materials and methods

### Data collection

The analyses were performed with data from the Amphia Cardiac Surgery Blood Management Study. Details of this study have been described earlier [13]. In this ongoing cohort study, peri-operative data are prospectively collected for all consecutive cardiac surgery patients since 1997. Data are collected in a distributed proprietary database during the complete peri-operative course. Variables regarding pre-operative co-morbidities, drug therapy, routine pre-, intra- and post-operative laboratory analysis, complications and post-operative outcome are collected at the Amphia Hospital in Breda, the Netherlands. The Amphia Hospital is a non-university hospital with the possibility of transferring special patient categories to tertiary hospitals.

All variables necessary to calculate both the ESI and the ESII are present in the database. After publication of the ESII the data dictionary was updated to reflect ESII additions and changes. The ESII update was implemented from April 2012 on. The database is compliant with the Dutch National Cardiac Surgery Registry and the Dutch National Intensive Care Registry [14].

Data collection took place between 1 April 2012 and 1 January 2014.

### Patient sample and analyses

All consecutive patients who underwent cardiac surgery were included regardless of the type of surgery. For each patient the additive and logistic ESI as well as the ESII were calculated using the formulas available at the EuroSCORE website (www.euroscore.org). The discriminative power of each risk model was assessed by plotting the receiver operating characteristic (ROC) curves of the different ES on in-hospital mortality, and comparing the area under the curve (AUC). The calibration of the different risk models was examined comparing the observed versus the expected values for in-hospital mortality rates. This way we could assess whether the ES (low or high) corresponded with the observed mortality. We performed the same analyses in subgroups according to type of surgery in four categories: coronary artery bypass graft (CABG), CABG combined with other surgery, valve surgery, miscellaneous procedures and according to the urgency of the procedure: emergency versus elective surgery. Emergency surgery was defined as surgery which had to be performed as soon as possible but within at least 24 h after admittance.

## Results

### Patient characteristics

Our cohort consisted of 2,296 patients; pre- and intra-operative patient characteristics are shown in Table 1. A total of 662 patients (28.8%) were female; the median age was 70 years (inter-quartile range (IQR) 63–76). The overall mortality in our cohort was 2.4% (55 patients).

**Table 1:** Patient characteristics (*n* = 2,296)

*Pre-operative variables* ^*a*^	*Missing*	
Age (years)	–	70 (63–76)
Female gender	–	662 (28.8)
Weight (kg)	–	80 (71–90)
Previous cardiac surgery	–	171 (7.4)
Previous myocardial infarction	–	311 (13.5)
NYHA class	–	
– I		942 (41.0)
– II		694 (30.2)
– III		484 (21.1)
– IV		176 (7.7)
LMCA >50% occluded	4	299 (13.0)
Left ventricular hypertrophy	24	536 (23.3)
Pulmonary artery pressure >40 mm Hg	–	58 (2.5)
Atrial fibrillation	1	399 (17.4)
Extra cardiac arteriopathy	–	311 (13.5)
Hypertension	8	1,445 (62.9)
Pre-operative Hb level (g/dl)	–	13.7 (12.6–14.8)
Creatinine level (µmol/l)	–	84 (72–100)
Creatinine clearance	–	80 (60–101)
Ejection fraction	–	
– good		1,758 (76.6)
– moderate		377 (16.4)
– poor		136 (5.9)
– very poor		25(1.1)
Smoking	19	366 (15.9)
Insulin-dependent diabetes	–	284 (12.4)
Endocarditis	–	28 (1.2)
Chronic renal failure^b^	–	50 (2.2)
COPD	–	267 (11.6)
Poor mobility	–	167 (7.3)
Pre-operative use of inotropic agents	–	25 (1.1)
Respiratory insufficiency	–	65 (2.8)
Jehovah’s witnesses	2	32 (1.4)
Emergency surgery	–	261 (11.4)
Aortic valve pathology	–	793 (34.5)
Mitral valve pathology	–	469 (20.4)
Type of surgery	–	
– CABG		1,059 (46.1)
– CABG + other surgery		319 (13.9)
– Valve		615 (26.8)
– Miscellaneous surgery		303 (13.2)
Off-pump procedure	–	139 (6.1)
Additive EuroSCORE I	–	5 (3–8)
Logistic EuroSCORE I	–	10.7 (5.8–13.9)
EuroSCORE II	–	1.6 (1.0–3.5)
*Intra-operative variables*	*Missing*	
Time in surgery (min)	11	215 (180–255)
CPB time (min)	–	83 (61–117)
Clamp time (min)	–	54 (36–78)
Intra-operative nadir Hb (g/dl)	15	9.7 (8.4–10.8)

*NYHA* New York Heart Association, *LMCA* left main coronary artery, *COPD* chronic obstructive pulmonary disease, *CABG* coronary artery bypass graft

^a^Presented as number (%) or median (inter-quartile range)

^b^Creatinine level above 177 mmol/l

### Performance of the EuroSCORE II

**Fig. 1:**
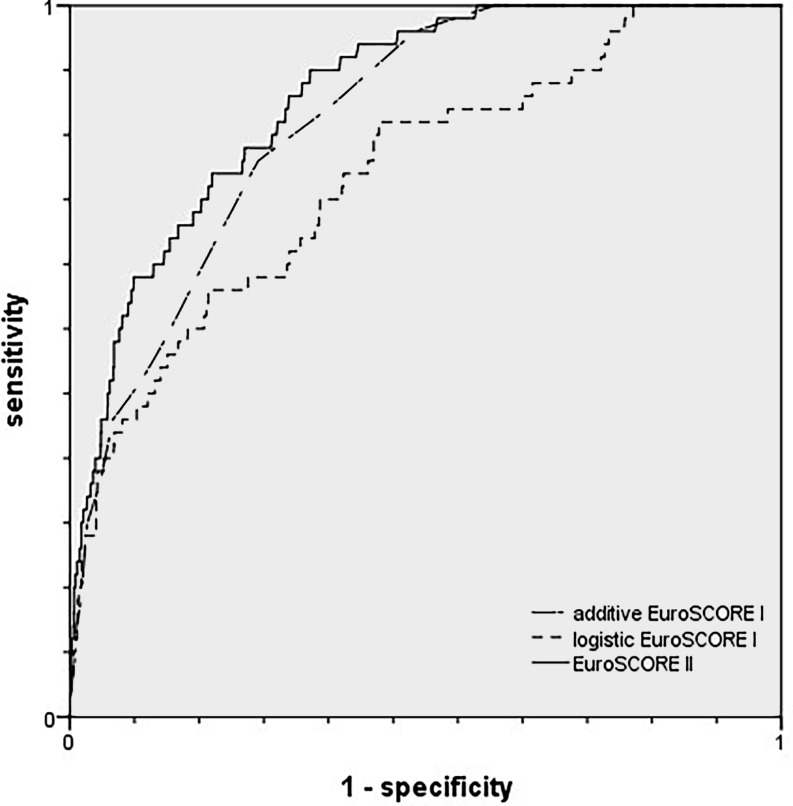
Receiver operating characteristic (ROC) curves for the additive and logistic EuroSCORE I and the EuroSCORE II, *n* = 2,269

We observed that although the discriminative power of both the additive as well as the logistic ESI was good, it was surpassed by the ES II (AUC additive ESI 0.840, 95% confidence interval (CI) 0.798–0.882; logistic ESI 0.761, CI 0.695–0.828; AUC ESII 0.871, CI 0.832–0.911). ROC curves are displayed in Fig. 1. The ESII underestimated observed mortality while the additive and the logistic ESI overestimated mortality (ESII observed versus expected (O/E) ratio 1.50 vs ESI additive 0.48 vs ESI logistic 0.22; results are displayed in Table 2).

**Table 2:** Discrimination and calibration parameters in total cohort (*n* = 2,296)

	Area under the curve	Observed mortality Number (%)	Expected mortality Median (IQR)	O/E ratio
Additive EuroSCORE I	0.840 (0.798–0.882)	55 (2.4)	5.0 (3.0–8.0)	0.48
Logistic EuroSCORE I	0.761 (0.695–0.828)	55 (2.4)	10.7 (5.8–13.9)	0.22
EuroSCORE II	0.871 (0.832–0.911)	55 (2.4)	1.6 (1.0–3.5)	1.50

*IQR* Inter-quartile range, *O/E* observed versus expected

### EuroSCORE II performance in different types of surgery

**Fig. 2:**
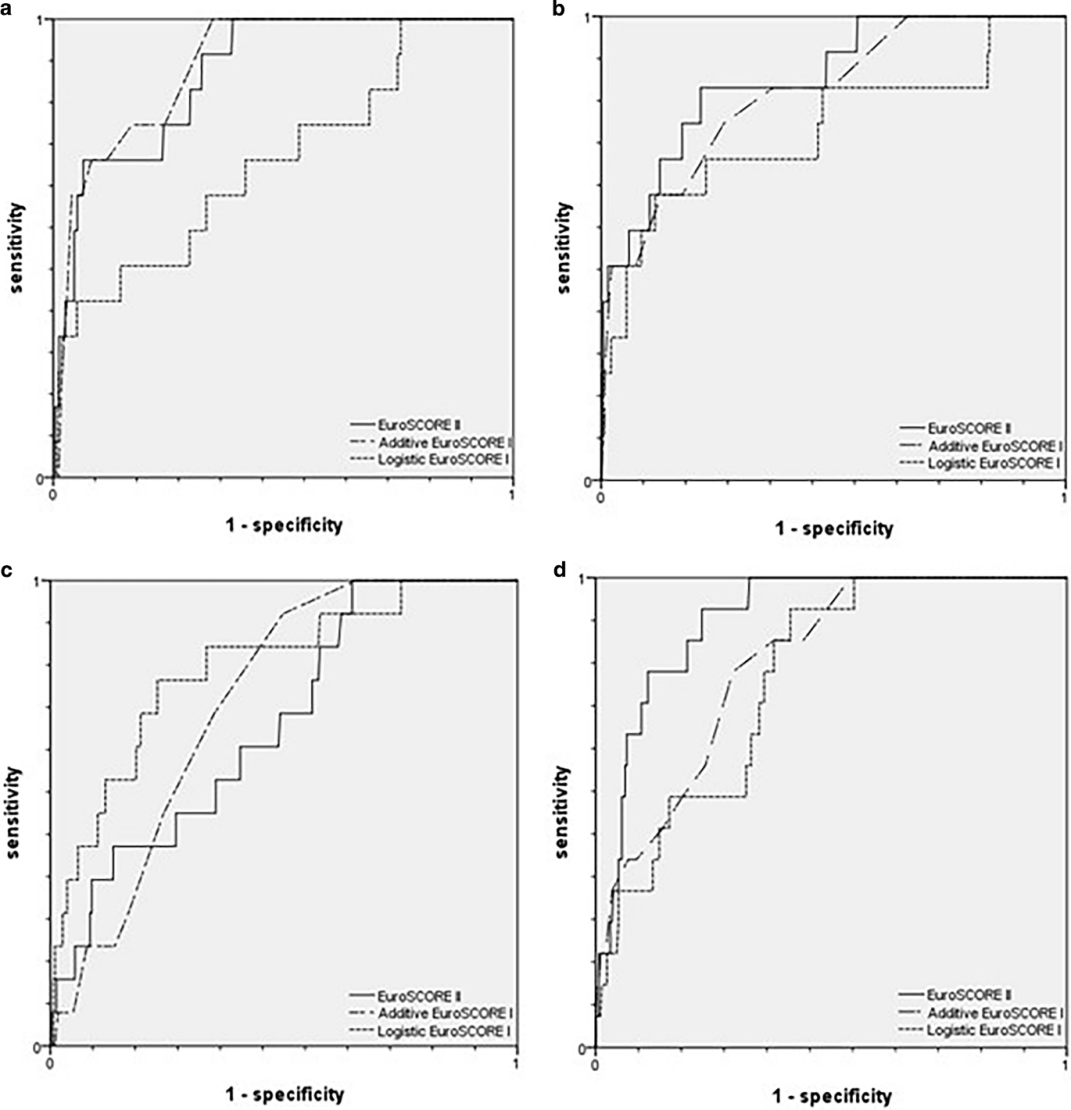
Receiver operating characteristic curves for the additive and logistic EuroSCORE I and the EuroSCORE II. **a** Patients undergoing a coronary artery bypass graft (CABG; *n* = 1,059). **b** Patients undergoing valve surgery (*n* = 615). **c** Patients undergoing a CABG in combination with any other procedure (*n* = 319). **d** Patients undergoing miscellaneous cardiac surgical procedures (*n* = 303)

A total of 1,059 patients underwent a CABG, 319 patients underwent a CABG in combination with another procedure, 615 patients underwent surgery involving one or more heart valves, and 303 patients underwent miscellaneous kinds of cardiac surgery. Table 3 shows the observed versus expected mortality for the different surgical procedures; the ROC curves are presented in Fig. 2.

**Table 3:** Observed versus expected mortality for the different surgical procedures

	Area under the curve	Observed mortality Number (%)	Expected mortality Median (IQR)	O/E ratio
*Discrimination and calibration according to surgical procedure*				
CABG (*n* = 1,059)				
– Additive EuroSCORE I	0.900 (0.838–0.962)	13 (1.2)	4.0 (3.0–6.0)	0.30
– Logistic EuroSCORE I	0.691 (0.536–0.846)	13 (1.2)	10.6 (4.8–13.7)	0.11
– EuroSCORE II	0.884 (0.809–0.958)	13 (1.2)	1.4 (0.93–2.6)	0.86
CABG + other procedure (*n* = 319)				
– Additive EuroSCORE I	0.739 (0.648–0.831)	14 (4.4)	7.0 (5.0–9.0)	0.63
– Logistic EuroSCORE I	0.812 (0.695–0.929)	14 (4.4)	10.3 (5.5–13.8)	0.43
– EuroSCORE II	0.693 (0.566–0.820)	14 (4.4)	3.4 (2.0–6.5)	1.29
Valve (*n* = 615)				
– Additive EuroSCORE I	0.833 (0.722–0.943)	13 (2.1)	7.0 (5.0–9.0)	0.30
– Logistic EuroSCORE I	0.755 (0.594–0.917)	13 (2.1)	11.2 (8.8–14.2)	0.19
– EuroSCORE II	0.866 (0.768–0.964)	13 (2.1)	1.5 (1.0–3.1)	1.40
Miscellaneous procedures (*n* = 303)				
– Additive EuroSCORE I	0.819 (0.732–0.907)	15 (5.0)	5.0 (2.0–9.0)	1.00
– Logistic EuroSCORE I	0.784 (0.692–0.876)	15 (5.0)	10.3 (4.5–14.2)	0.49
– EuroSCORE II	0.912 (0.862–0.962)	15 (5.0)	1.5 (0.7–4.7)	3.33
*Discrimination and calibration according to urgency*				
Elective surgery (*n* = 2,035)				
– Additive EuroSCORE I	0.827 (0.779–0.875)	30 (1.5)	5.0 (3.0–7.0)	0.30
– Logistic EuroSCORE I	0.720 (0.621–0.818)	30 (1.5)	10.5 (5.2–13.7)	0.14
– EuroSCORE II	0.839 (0.784–0.894)	30 (1.5)	1.5 (0.92–2.9)	1.00
Emergency surgery (*n* = 261)				
– Additive EuroSCORE I	0.726 (0.616–0.836)	25 (9.6)	10.0 (7.0–13.0)	0.96
– Logistic EuroSCORE I	0.729 (0.631–0.826)	25 (9.6)	12.9 (9.2–17.5)	0.74
– EuroSCORE II	0.816 (0.736–0.896)	25 (9.6)	5.9 (2.2–13.2)	1.63

*CABG* Coronary artery bypass graft, *IQR* inter-quartile range, *O/E* observed versus expected

In patients who underwent a CABG in-hospital mortality was 1.2%. The additive ESI had the best discriminative power (AUC 0.900, CI 0.838–0.962) and the ESII was the best calibrated risk model (O/E ratio ESII 0.86). In patients who underwent a CABG in combination with other surgery in-hospital mortality was 4.4%. The discriminative power of the logistic ESI was highest (AUC 0.812, CI 0.695–0.929). Although mortality was underestimated by the ESII, the ESI overestimated the mortality even more. Therefore the ESII provided a better calibration (O/E ratio ESII 1.29).

In the patients who underwent isolated valve surgery the mortality was 2.1%. The ESII had the highest discriminative power and was better calibrated (AUC 0.866, CI 0.768–0.964, O/E ratio 1.40). In patients who underwent miscellaneous cardiac surgery, mortality was 5.0%. The ESII showed the best discrimination (AUC 0.912, CI 0.862–0.962). The best calibration was performed by the additive ESI (O/E ratio 1.00).

### EuroSCORE II performance in emergency surgery

**Fig. 3:**
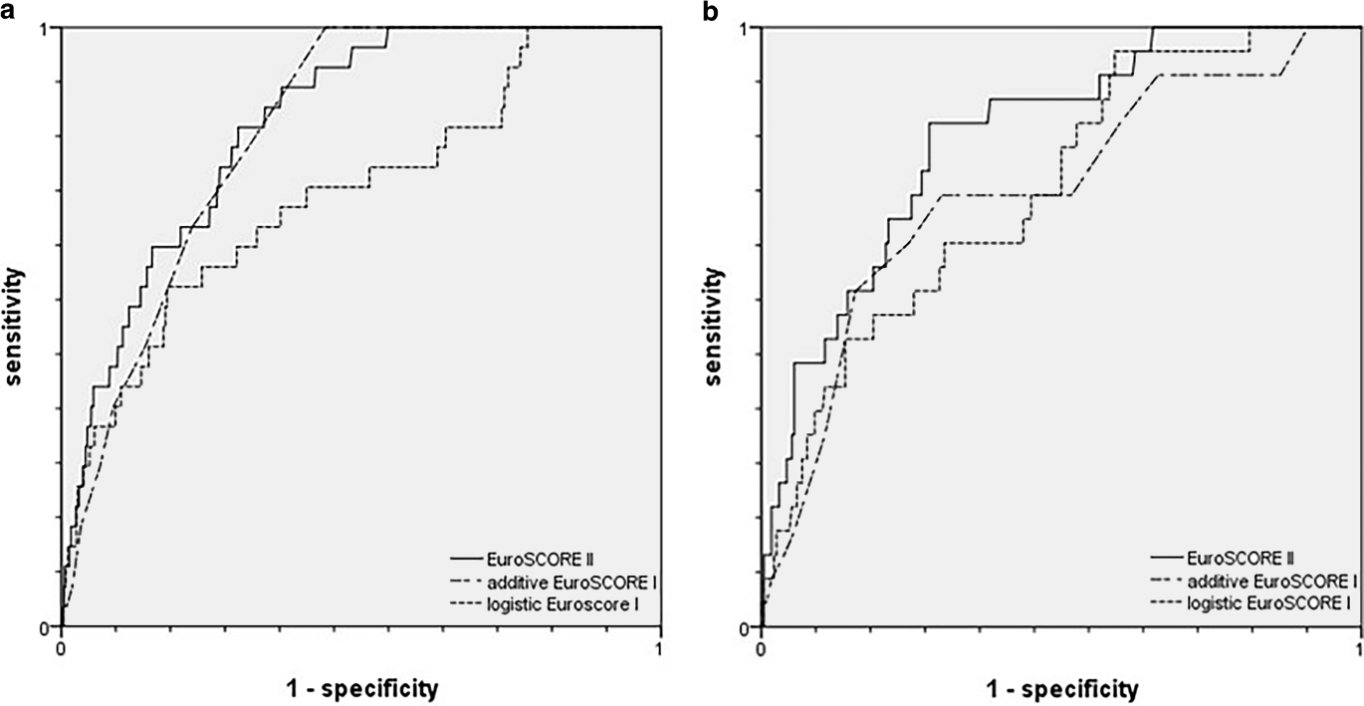
Receiver operating characteristic curves for the additive and logistic EuroSCORE I and the EuroSCORE II. **a** Patients undergoing elective surgery (*n* = 2,035). **b** Patients undergoing emergency surgery (*n* = 261)

A total of 261 patients underwent emergency surgery and 2,035 patients underwent elective surgery. Table 3 shows the observed versus expected mortality in patients who underwent elective or emergency surgery (ROC curves are displayed in Fig. 3). In patients who underwent elective surgery the ESII had the highest discriminative power and was best calibrated (AUC 0.839, CI 0.784–0.894, O/E ratio1.00). In patients who underwent emergency surgery the ESII had the highest discriminative power, but the additive ESI was better calibrated (ESII: AUC 0.816, CI 0.736–0.896, O/E ratio 1.63; ESI: AUC 0.726, CI 0.616–0.836, O/E ratio 0.96).

## Discussion

### Main findings

In this validation study, we found that the ESII is a well-calibrated risk model with a good predictive value. The ESII underestimated the mortality in some subgroups, while the ESI overestimated the observed mortality. Nevertheless the expected mortality of the ESII was closer to the observed mortality than was the case for the ESI; therefore the ESII outperformed the ESI in most patients. Whether this is also true for patients who underwent CABG surgery in combination with another procedure, miscellaneous surgery or emergency surgery in this study is not certain due to the small sample size of the subgroups.

### Limitations and strengths

A limitation of our study is that it is a single-centre study, which could impair the generalisability of our results. There is little reason to believe that the patient population at the Amphia Hospital will differ greatly from those of other peripheral hospitals in the Netherlands. Nevertheless, bias due to population differences could not be ruled out. Furthermore, the sample sizes of some subcategories used in our subgroup analyses (CABG in combination with another procedure, miscellaneous surgery and emergency surgery) might be too small to exclude random error. Also, validating the ESII for 30-day and 90-day mortality was not possible with our present data.

Strengths of this validation study are that our data were collected prospectively after the implementation of the ESII in clinical practice. The database used is accurate, and very few data are missing.

In order to validate a risk model like the ESII, validation studies must satisfy certain requirements. In this study we avoided pitfalls which could lead to biased results. For example, the ESII was developed based on data collected during a 12-week period in 2010 and was intended for prospective use [1]. Some studies have validated the ESII based on data acquired before 2010, and some studies included patients over an extended period of time. This could lead to biased results because of changes and improvements made in daily practice [15]. An example of this bias is illustrated in a study that validates the ESII in two different time periods (R.L. Osnabrugge et al. 2012, conference abstract). First, the ESII was validated in patients who had surgery between 2003 and 2012. The results from the analyses showed that although the ESII had the highest discriminative power, the STS score had a better calibration. After this analysis, the ESII was validated in a subgroup of patients who had surgery between 2008 and 2012. Results from these analyses show that the ESII (based on patient data collected in 2010) was the best overall risk model. Also, there are studies in which the ESII has been validated in surgical procedures for which the ESII was never intended (for example in transcatheter aortic valve implantation). This also could lead to suboptimal performance [16]. Third, some validation studies present incomplete data or use inaccurate statistical methods: for example, basing the conclusions solely on a non-significant Hosmer-Lemeshow test, instead of showing the complete data [17]. In our present study we avoided these known pitfalls.

### Comparison with other EuroSCORE II validation studies

The observed in-hospital mortality in our study (2.4%) differed slightly from the overall mortality of cardiac surgery procedures in the Netherlands (3.0%) or in neighbouring countries like the UK (2.7%) [17, 18]. Baseline characteristics of our cohort and the patient sample on which the original ESII was based were nearly identical. The only variable that differed between our study and the original sample was insulin-dependent diabetes (12.4% in our cohort versus 7.6%). The mortality rates of the original patient sample (3.9%) and the mortality rates in our cohort differed by 1.5%.

Ever since its publication in 2012, more than 50 studies concerning the ESII have been published with varying results. Our results agree with a majority of these studies [19–23]. Two studies also analysed the performance of the ESII according to type of surgery using the same categories we used. Curiously, although the ESII performed well, these studies found that the ESI was the overall best performing risk model in elective surgery [24, 25]. This is due to a higher mortality rate than in our cohort. Why the mortality of these two cohorts differs from ours is not precisely known, but these differences could be due to different population characteristics such as New York Heart Association class and gender.

In a previous study focussing solely on the performance of the ESII in patients who had emergency surgery, the ESII outperformed the ESI, although both showed poor calibration and discrimination [26]. In our cohort the overall performance of the models was worse in patients who underwent emergency surgery than in those who did not. In our cohort of emergency patients the ESII had a good discriminative power but a rather poor calibration, while the additive ESI had a good calibration. It is possible that other logistic factors, e. g. time to diagnosis or time required to get a patient to a specialised centre, play a more important role in the risk evaluation of emergency patients than factors included in the ES.

One study retrospectively evaluated the performance of the ESII in the Netherlands with similar results to ours [23]. However, this was a small study examining only 100 patients who underwent CABG surgery combined with mitral or aortic valve surgery.

### Implications for practice and future research

Before implementing a risk model in daily practice it is important to externally validate the model in local populations [5]. Our findings show that the ESII is a good risk predictor which outperforms its predecessor. Based on the results in our cohort we recommend using the ESII as the standard tool for risk prediction in patients undergoing cardiac surgery. A significant proportion of our cohort consisted of patients undergoing an isolated CABG with relatively short cardiopulmonary bypass (CPB) times. Therefore we also recommend that all hospitals using or planning to use ESII should validate the ESII in their setting because, for example, differences in CPB time or operation type could influence the predictive value of the ESII.

Another topic for future research should be identifying other variables that could increase the accuracy of the ESII, especially with regard to emergency surgery and/or high-risk patients because it seems the ESII has the least predictive value in those populations.

In conclusion, the ESII is a good predictive model of short-term mortality in cardiac surgery. It is better calibrated than its predecessor, the ESI, in patients undergoing elective surgery.
